# Psychopathological symptoms as precursors of depressive symptoms in adolescence: a prospective analysis of the GINIplus and LISA birth cohort studies

**DOI:** 10.1007/s00127-022-02267-1

**Published:** 2022-04-15

**Authors:** Ellen Greimel, Lena Adams, Carolin Zsigo, Dietrich Berdel, Andrea von Berg, Sibylle Koletzko, Carl-Peter Bauer, Tamara Schikowski, Gunda Herberth, Joachim Heinrich, Gerd Schulte-Körne, Marie Standl

**Affiliations:** 1 Department of Child and Adolescent Psychiatry, Psychosomatics and Psychotherapy, Hospital of the Ludwig-Maximilians-University (LMU) Munich, Waltherstr. 23, 80337 Munich, Germany; 2 Department of Pediatrics, Research Institute, Marien-Hospital Wesel, Wesel, Germany; 3 Department of Pediatrics, Dr. von Hauner Children’s Hospital, University Hospital, LMU Munich, Munich, Germany; 4 Department of Pediatrics, Gastroenterology and Nutrition, School of Medicine Collegium Medicum University of Warmia and Mazury, Olsztyn, Poland; 5 Department of Pediatrics, Technical University of Munich, Munich, Germany; 6 IUF-Leibniz Research Institute for Environmental Medicine, Düsseldorf, Germany; 7 Department of Environmental Immunology, Helmholtz Centre for Environmental Research-UFZ, Leipzig, Germany; 8 Institute of Epidemiology, Helmholtz Zentrum München-German Research Centre for Environmental Health, 85764 Neuherberg, Germany; 9 Institute and Clinic for Occupational, Social and Environmental Medicine, University Hospital, Ludwig Maximilians University of Munich, Munich, Germany; 10 Allergy and Lung Health Unit, Centre for Epidemiology and Biostatistics, School of Population and Global Health, The University of Melbourne, Melbourne, Australia

**Keywords:** Depressive symptoms, Adolescence, Strengths and Difficulties Questionnaire, Prospective analysis, Epidemiology

## Abstract

**Introduction:**

Depressive symptoms are highly prevalent in adolescence, highlighting the need for early identification of precursors. Research into psychopathological symptoms predicting depressive psychopathology in adolescents is therefore of great relevance. Moreover, given that the prevalence of depressive symptomatology in adolescence shows marked differences between girls and boys, insight into potential sex-specific differences in precursors is important.

**Methods:**

This study examined the relationships between emotional problems, conduct problems, hyperactivity/inattention, peer problems, and difficulties in prosocial behaviour at age 10 (Strengths and Difficulties Questionnaire), and the presence of depressive symptoms at age 15 (Depression Screener for Teenagers). Using data from 2824 participants of the GINIplus and LISA birth cohorts, the association of each SDQ subscale at age 10 years with the presence of depressive symptoms at age 15 years was analyzed using sex-specific logistic regression, adjusting for potential confounders.

**Results:**

Emotional problems [odds ratio (OR) 1.99, *p* = 0.002 for boys and OR 1.77, *p* < 0.001 for girls] and peer problems (OR 2.62, *p* < 0.001 for boys, OR 1.91, *p* = 0.001 for girls) at age 10 showed an increased risk for the presence of depressive symptoms at age 15. Additionally, boys with conduct problems at age 10 were at greater risk of showing depressive symptoms in adolescence (OR 2.50, *p* < 0.001).

**Discussion:**

Based on the identified prospective relationships in our study, it might be of particular importance to tailor prevention approaches during childhood to peer and emotional problems to reduce the risk of depressive psychopathology in adolescence. Moreover, particularly in boys, it seems important to also target conduct problems in childhood as a precursor of depressive symptoms in the adolescent period.

**Supplementary Information:**

The online version contains supplementary material available at 10.1007/s00127-022-02267-1.

## Introduction

Major depression (MD) is among the most debilitating mental disorders worldwide [1]. During adolescence, the risk of suffering from a depressive episode sharply rises to a 12-month prevalence rate of about 7.5%, with higher rates in girls compared to boys [2]. Depression during adolescence is associated with severe consequences, including increased suicide rates, psychosocial and (mental) health problems in adulthood [3].

Subclinical depressive symptoms without a diagnosis of MD are also frequent in adolescents, with an estimated 12-month prevalence rate between 4–9% and a higher preponderance in girls ([4], for a review, see [5]). Their presence is a strong predictor for a major depressive episode later in life and increases the risk of later comorbidity and psychosocial impairment [6–8]. Thus, it is pivotal to gain insight into antecedents of elevated depressive symptoms in adolescence to identify specific targets for early preventative approaches.

Several longitudinal studies have investigated which psychopathological symptoms predict depressive symptomatology in adolescence. Most have focused on a specific domain of psychopathology, e.g., conduct or peer problems (for a meta-analysis, see [9]). However, singular predictors can only contribute to a small part of the variance in depressive symptomatology (e.g., [10–13]) and are thus limited in their predictive value. Examining multiple predictors at the same time can enhance the ability to predict later depressive psychopathology. Such a wide-spanning approach should encompass a variety of psychopathological symptoms and problems youths often face before and during adolescence. This includes depressed mood and anxiety as well as conduct problems (involving aggressive, oppositional and antisocial behavior), inattention/hyperactivity, and difficulties with peer relationships [14–16]. In what follows next, we provide a detailed rationale for the relevance of these domains:

Depressive symptoms and negative emotionality show a marked continuity in youth [17] and predict elevated depressive symptoms in adolescents [18, 19]. Similarly, anxious symptoms, including excessive worry and oversensitivity, have been identified as a predictor for adolescent depressive symptomatology [18], especially in girls [20]. This can be brought in line with research suggesting that depressive and anxiety disorders share common etiological factors [21] as well as similarities in clinically manifest symptoms [22, 23].

Problematic conduct in children has similarly been shown to be a precursor of depressive symptoms in adolescence [9]. Individuals who show physical or indirect aggression in late childhood are at an increased risk for elevated depressive symptoms in adolescence, and early-onset conduct problems have a strong predictive value for a depressive episode in adolescence [24, 25]. It has been claimed that the comorbidity between conduct problems and depressive symptoms is higher in girls than in boys [26, 27]. However, other research did not find sex differences in this association [25], and it is unclear whether there are sex differences in the longitudinal relationship between conduct problems and depressive symptoms. Finally, looking at subclinical symptoms of ADHD, one study found a connection between ADHD symptoms in youth and higher levels of internalizing problems at age 19–20 [28]. Beyond this, a diagnosis of ADHD in childhood predicts both elevated depressive symptoms and a diagnosis of MD in adolescence [12, 29, 30].

In the transition phase from childhood to adolescence, peer relationships gain importance [31, 32] and peers become an increasingly important source of social support outside the family [33, 34]. Problems with one’s peers, however, can be a critical source of stress which predisposes adolescents to depressive psychopathology [35]. In this context, studies have demonstrated that troubles in peer relationships, including experiences of rejection and loneliness, predict depressive symptomatology in adolescent boys and girls alike.

To date, insight into sex differences in prospective associations of psychopathological symptom complexes with depressive symptoms in youth is scarce. However, this aspect is relevant for several reasons: First, girls experience a sharper rise in the prevalence of depressive symptoms during adolescence [38]. Second, girls and boys show different patterns of comorbidities: Girls tend more towards comorbid depression and anxiety, while boys tend towards depression and conduct problems [39]. Finally, some precursors of depression are influenced by sex. For example, peer problems have been found to be an independent risk factor for depressive psychopathology in boys, but not in girls (for a review, see [31]). Given this, sex-specific differences may also exist in precursors of depressive symptoms. Insight into such differences could be considered when tailoring prevention for depressive psychopathology, for example by putting a different emphasis on certain aspects of prevention programs in girls and boys, respectively.

While previous findings on longitudinal relationships between specific domains of psychopathology and depressive symptoms in adolescence provide important insights, studies are needed that examine multiple possible precursors to determine both their relative and combined predictive value. Such an approach is especially important in the transition between childhood and adolescence, as prevalence rates substantially increase in early adolescence [2]. Identifying precursors in late childhood offers the potential of targeted prevention efforts before depressive psychopathology manifests in vulnerable individuals.

The aims of the present study were thus to (1) investigate prospective associations between several psychopathological symptom domains and the presence of depressive symptoms in adolescence, (2) thereby examining boys and girls separately to identify sex-specific patterns. Depressive symptoms were assessed at age 15 and related to psychopathological symptoms at the age of 10 years based on prospectively collected data from two large population-based birth cohorts. Contrasting most previous studies with a narrower focus, we applied a broadband screening instrument for psychopathology covering emotional problems (encompassing anxious/depressed symptoms), conduct problems, hyperactivity/inattention, peer problems, and difficulties in prosocial behavior. Based on previous findings (e.g., [9, 20]), we expected that (a) increased emotional, conduct and peer problems at the age of 10 would predict the presence of depressive symptoms at the age of 15. We also expected that (b) the prospective association between peer problems and depressive psychopathology would be stronger in boys (see [31]). Furthermore, based on sex-specific comorbidity patterns [39], we hypothesized that (c) depressive symptoms are more strongly associated with conduct problems in boys and emotional problems in girls.

## Methods

### Study population

The present study is based on data obtained from the GINIplus (German Infant Nutritional Intervention plus environmental and genetic influences on allergy development) and LISA (Influence of Life-style related factors on the development of the Immune System and Allergies in East and West Germany) birth cohort studies. Both studies are population-based birth cohorts comprising healthy, term-born infants. The aim of both cohorts is to investigate the natural course of common chronic disease, with a focus on allergic diseases and related comorbidities as well as the identification of environmental and lifestyle factors associated with disease development. In the GINIplus study, 5991 participants were recruited between 1995 and 1998 from the two German regions Wesel and Munich [40].

The GINIplus consists of an observation arm and an intervention arm. For the intervention arm, newborns with a family history of allergic diseases were invited and randomized to receive one of three hydrolyzed formulas or cow’s milk with the aim to compare the effect of the different formulas on allergy development [41]. Participants who declined to participate in the intervention trial and children without a family history of allergic diseases were included in the observation arm. The LISA birth cohort includes 3094 participants from Munich, Wesel, Leipzig and Bad Honnef born from 1997 to 1998 [42].

The present analysis is based on data collected during the 10- and 15-year follow-ups (Supplementary Figure S6). Both studies were approved by the local ethics committees. All participants were informed in detail about the procedures and the aims of the study, and provided written informed assent. Moreover, written informed consent was obtained from at least one legal guardian, after the legal guardian(s) had been informed about all aspects of the study.

### The Strength and Difficulties Questionnaire (SDQ)

The Strength and Difficulties Questionnaire (SDQ) is a brief screening questionnaire assessing psychopathological symptoms, including emotions, behaviors and social relationships in children and adolescents [14]. The questionnaire was administered to the parents during the 10-year follow-up [43, 44].

The SDQ contains 25 items, which are divided into five subscales (emotional problems, conduct problems, hyperactivity/inattention, peer problems, and prosocial behavior) [14]. The subscale prosocial behavior is inverted so that higher values represent more difficulties. The sum of the scores of the four subscales emotional problems, conduct problems, hyperactivity/inattention and peer problems generate a total difficulties score. The SDQ total scale and the subscales of both the self-report and the parent version have shown good to satisfactory reliability [45, 46].

Following official scoring guidelines (www.sdq.info), SDQ scores of the subscales were categorized into three levels, “normal”, “borderline” and “abnormal”, applying existing German cut-offs [46, 47]. For statistical analyses, a dichotomous variable was created for each SDQ subscale, in which the “normal” respondents were compared to “borderline/abnormal” respondents [43].

### The Depression Screener for Teenagers (DesTeen)

The Depression Screener for Teenagers (DesTeen) was used to assess the presence of depressive symptoms [48]. It was administered to the participants during the 15-year follow-up. The DesTeen is a self-report screening questionnaire for depressive symptoms in adolescents aged 13–16 years old. The DesTeen has shown good reliability [49]. It comprises 14 items that are answered on a four-point Likert scale and assess cognitive and emotional symptoms of depression over the preceding 2 weeks [49, 50].

Presence of depressive symptoms was defined as a total score ≥ 12, which has been shown as the ideal cut-off for any depressive disorder (including major depression, dysthymia and minor depression as defined by DSM-IV-TR criteria [51]) based on a validation study in a pediatric sample [49]. In an additional sensitivity analysis, the presence of depressive symptoms was defined as a total score ≥ 14 which is the recommended cut-off to screen for major depression and dysthymia (excluding cases of minor depression) based on DSM-IV-TR criteria ([50], see also [49, 52]). As (1) validation data for the cut-off ≥ 14 are not publicly available, and (2) the present study focused on the presence of depressive symptoms rather than clinically relevant diagnoses, our main analyses were based on the validated cut-off ≥ 12.

### Confounder variables

All analyses were adjusted for study (GINIplus observation arm/GINIplus intervention arm/LISA), study region (Munich/Leipzig/Bad Honnef/Wesel), exact age when filling out the DesTeen during the 15-year follow-up, parental education defined by the highest grade completed by either the mother or the father on the basis of the German educational system (low < 10th grade/medium = 10th grade/high > 10th grade), single-parent household at the 15-year follow-up and pubertal stage based on a self-rating pubertal development scale [53, 54] (dichotomized into early/mid pubertal and late/post-pubertal). Single-parent household was controlled for because it is seen *as a proxy* for several disadvantages, such as family living standards and family problems [55], which were not assessed separately.

In a sensitivity analysis, the main regression model was additionally adjusted for parental psychopathology (yes/no). Parental psychopathology was defined as cases with a Global Severity Index score (from the Brief Symptom Inventory 18 [56]) greater than the 90th percentile to ensure a sufficient number of cases, as there are no published reference values for the German population.

### Statistical analysis

Given well-documented differences in the prevalence of depressive symptoms between sexes [57], all analyses were conducted separately for males and females. Descriptive statistics are presented as mean with standard deviation (SD) for continuous variables and counts and percentages for categorical variables. Differences between males and females were tested using *t* test for continuous variables, Fisher’s exact test for binary variables and Chi-squared test for categorical variables with more than two categories. Due to the skewed distribution of the sum scores, SDQ subscales and depressive symptoms assessed using DesTeen were dichotomized to enable comparability between both questionnaires and simplify interpretation. Logistic regression was used to determine the effect of the selected confounding variables.

Further, the association of each SDQ subscale at ages 10 years with presence of depressive symptoms at age 15 years was analyzed using logistic regression, adjusting for study, study region, age, parental education level, single-parent household and pubertal stage. Significant sex differences were tested in an additional model in which an interaction term of the SDQ subscale with sex was included. Several sensitivity analyses were conducted: (1) main logistic regression models were further adjusted for parental psychopathology; (2) all SDQ subscales were included in one model for mutual adjustment and (3) presence of depressive symptoms was defined with a stricter cut-off ≥ 14 (instead of ≥ 12) and (4) including the SDQ scales using a three-level categorization (normal, borderline and abnormal with normal being the reference category). Results of the logistic regression models are presented as adjusted odds ratio (OR) with a corresponding 95% confidence interval (95% CI). As all regression models were conducted stratified by sex; Bonferroni correction for multiple testing was applied to the logistic regression models and the *α*-level was divided by 2 (corrected *p*-value threshold for significance: 0.05/2 = 0.025). All analyses were conducted using R, version 3.5.1 [58].

## Results

Of 4926 subjects participating in the 15-year follow-up, 3989 participants returned the completed DesTeen questionnaire with no missing values (Supplementary Figure S6). Of these 3989 participants, 3505 participants had information on at least one SDQ subscale at 10 years. Complete information on all confounders (study, study region, age, parental education level, single-parent household and pubertal stage) was available for 2824 participants (1456 females and 1368 males) who were included in the analysis (Table 1).

**Table 1:** Study population characteristics

	Females (*n* = 1456)	Males (*n* = 1368)	*p*-value^a^
Age at 15-year follow-up	15.2 (0.3)	15.1 (0.3)	0.2138
Pubertal stage at 15-years (late/post vs. early/mid pubertal)	1387/1456 (95.3%)	803/1368 (58.7%)	< 0.0001
Study center			0.8830
Munich	750/1456 (51.5%)	725/1368 (53%)	
Leipzig	144/1456 (9.9%)	133/1368 (9.7%)	
Bad Honnef	62/1456 (4.3%)	55/1368 (4%)	
Wesel	500/1456 (34.3%)	455/1368 (33.3%)	
Single parent household at 15-year follow-up	192/1456 (13.2%)	196/1368 (14.3%)	0.3822
Parental education level			0.1207
Low	71/1456 (4.9%)	85/1368 (6.2%)	
Medium	365/1456 (25.1%)	368/1368 (26.9%)	
High	1020/1456 (70.1%)	915/1368 (66.9%)	
Parental psychopathology	165/1443 (11.4%)	133/1353 (9.8%)	0.1776
Study			0.1139
GINIplus observation	559/1456 (38.4%)	485/1368 (35.5%)	
GINIplus intervention	381/1456 (26.2%)	348/1368 (25.4%)	
LISA	516/1456 (35.4%)	535/1368 (39.1%)	
Presence of depressive symptoms at age 15 (DesTeen)^b^	253/1456 (17.4%)	136/1368 (9.9%)	< 0.0001
Borderline/abnormal scores in SDQ scales at age 10^c^			
Total difficulties	141/1456 (9.7%)	238/1367 (17.4%)	< 0.0001
Emotional problems	242/1456 (16.6%)	221/1367 (16.2%)	0.7604
Conduct problems	118/1456 (8.1%)	184/1368 (13.5%)	< 0.0001
Hyperactivity/inattention	110/1456 (7.6%)	241/1367 (17.6%)	< 0.0001
Peer problems	89/1456 (6.1%)	129/1367 (9.4%)	0.0011
Prosocial behavior^d^	67/1456 (4.6%)	129/1367 (9.4%)	< 0.0001

Values presented as *n*/*N* (%) or mean (SD)

^a^*p*-values were obtained from *t* test for continuous variables, Fisher’s exact test for binary variables and Chi-squared test for categorical variables with more than two categories

^b^Presence of depressive symptoms was defined as a total score ≥ 12 in the Depression Screener for Teenagers (DesTeen)

^c^For each scale of the Strengths and Difficulties Questionnaire (SDQ), a dichotomous variable was created. The numbers represent the number and percentage of “borderline/abnormal” respondents

^d^Borderline/abnormal scores in this scale refer to difficulties in prosocial behavior

The population included in the present analysis differed from the original study population showing lower prevalences of SDQ total difficulties as well as the SDQ subscales emotional problems, conduct problems, hyperactivity/inattention and peer problems (Supplementary Table S1). Furthermore, the participants included in the analysis were more likely to be female, younger, from the Munich study center, not living in a single-parent household, having a higher parental education level and participating in the GINI intervention arm or LISA study.

Females reported the presence of depressive symptoms significantly more often than males (females 17.4%, males 9.9%, *p* < 0.0001; Table 1). Significantly more males than females had borderline or abnormal scores in all SDQ subscales (all *p*-values ≤ 0.0011), except for emotional problems where no significant differences were observed (*p* = 0.7604). Females presented the highest prevalence (i.e., borderline/abnormal scores) for emotional problems (16.6%), while hyperactivity/inattention showed the highest prevalence in males (17.6%). Significantly more females than males were late or post pubertal (95.3% vs. 58.7%, *p* < 0.0001). Study center, study, age, single-parent household and parental education level did not differ significantly between males and females (Table 1).

The effect of the confounding variables on depressive symptoms is presented in Table 2. In females, only higher age was significantly associated with the presence of depressive symptoms (OR 1.79 per year, *p* = 0.0115). In males, the prevalence of depressive symptoms was significantly higher in participants from Leipzig compared to participants from Munich (OR 2.06, *p* = 0.0158). Furthermore, participants whose parents had medium (OR 0.40, *p* = 0.0083) or high (OR 0.34, *p* = 0.0010) education level (compared to low education level) reported fewer depressive symptoms.

**Table 2:** Results of logistic regression of confounding variables on depressive symptoms

	OR	95% CI	*p*-value
(a) *Females*			
Age at 15-year follow-up	1.79	(1.13; 2.8)	0.0115
Pubertal stage at 15 years (late/post vs. early/mid pubertal)	1.43	(0.71; 3.3)	0.3497
Study center (Leipzig vs. Munich)	1.24	(0.74; 2.03)	0.4077
Study center (Bad Honnef vs. Munich)	0.63	(0.27; 1.30)	0.2338
Study center (Wesel vs. Munich)	0.81	(0.58; 1.14)	0.2335
Single parent household	0.79	(0.55; 1.18)	0.2363
Parental education level (medium vs. low)	0.88	(0.46; 1.78)	0.7084
Parental education level (high vs. low)	0.84	(0.46; 1.64)	0.5806
Study (GINIplus intervention vs. GINIplus observation)	1.07	(0.75; 1.53)	0.7006
Study (LISA vs. GINIplus observation)	1.23	(0.84; 1.81)	0.2822
(b) *Males*			
Age at 15 year follow-up	1.29	(0.66; 2.46)	0.4457
Pubertal stage at 15 years (late/post vs. early/mid pubertal)	0.84	(0.58; 1.22)	0.3578
Study center (Leipzig vs. Munich)	2.06	(1.14; 3.70)	0.0158
Study center (Bad Honnef vs. Munich)	1.47	(0.60; 3.24)	0.3658
Study center (Wesel vs. Munich)	0.69	(0.42; 1.11)	0.1332
Single parent household	0.93	(0.58; 1.57)	0.7855
Parental education level (medium vs. low)	0.40	(0.21; 0.81)	0.0083
Parental education level (high vs. low)	0.34	(0.18; 0.66)	0.0010
Study (GINIplus intervention vs. GINIplus observation)	1.21	(0.72; 2.01)	0.4714
Study (LISA vs. GINIplus observation)	1.38	(0.82; 2.32)	0.2235

Presence of depressive symptoms at age 15 was defined as a total score ≥ 12 in the Depression Screener for Teenagers (DesTeen). Bonferroni-corrected *p*-value = 0.025

**Fig. 1:**
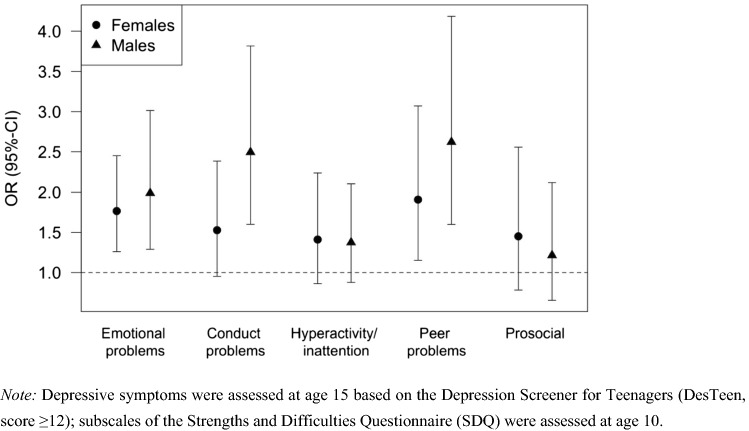
Results of logistic regression analysis regressing each SDQ subscale separately on depressive symptoms, adjusted for the confounding factors presented in Table 2. Note: Depressive symptoms were assessed at age 15 based on the Depression Screener for Teenagers (DesTeen, score ≥ 12); subscales of the Strengths and Difficulties Questionnaire (SDQ) were assessed at age 10

Results of the logistic regression analysis are presented in Table 3 and Fig. 1. Females with emotional problems (OR 1.77, *p* = 0.0008) or peer problems (OR 1.91, *p* = 0.0096) at age 10 showed an increased risk for the presence of depressive symptoms at age 15. In males, emotional problems (OR 1.99, *p* = 0.0015), conduct problems (OR 2.50, *p* < 0.0001) and peer problems (OR 2.62, *p* = 0.0001) at age 10 were associated with an increased risk for the presence of depressive symptoms at age 15. No significant sex-specific differences were found (Fig. 1). These results did not change substantially when further adjusting for parental psychopathology (Supplementary Table S2). The effect estimates were slightly attenuated and the *p*-values higher, but still significant, except the association of peer problems with the presence of depressive symptoms in females, which did not reach significance after adjustment for multiple testing (OR 1.75, 95% CI (1.04; 2.87), *p* = 0.0292).

**Table 3:** Results of logistic regression analysis regressing each SDQ subscale separately on depressive symptoms, adjusted for the confounding factors presented in Table 2

	Females			Males		
	OR	95% CI	*p*-value	OR	95% CI	*p*-value
Emotional problems	1.77	(1.26; 2.45)	0.0008	1.99	(1.29; 3.01)	0.0015
Conduct problems	1.53	(0.95; 2.38)	0.0689	2.50	(1.60; 3.82)	< 0.0001
Hyperactivity/inattention	1.41	(0.86; 2.24)	0.1561	1.37	(0.88; 2.10)	0.1526
Peer problems	1.91	(1.15; 3.07)	0.0096	2.62	(1.60; 4.18)	0.0001
Prosocial behavior	1.45	(0.78; 2.56)	0.2151	1.21	(0.65; 2.12)	0.5135

Depressive symptoms were assessed at age 15 based on the Depression Screener for Teenagers (DesTeen, score ≥ 12); subscales of the Strengths and Difficulties Questionnaire (SDQ) were assessed at age 10. Bonferroni-corrected *p*-value = 0.025

When mutually adjusting for all SDQ subscales in the same model (Supplementary Table S3), only emotional problems at age 10 years were significantly associated with the presence of depressive symptoms in females, while in males the significant association of conduct problems and peer problems at age 10 with elevated depressive symptoms remained, although the effect size was slightly attenuated.

In a sensitivity analysis, depressive symptoms were defined using a higher cut-off of 14 instead of 12 (Supplementary Table S4). The results were generally comparable with the main analysis. Additionally, the association of hyperactivity/inattention at age 10 years with depressive symptoms reached significance in males (OR 1.94, *p* = 0.0121).

Furthermore, the sensitivity analysis analyzing the SDQ scales using a three-level categorization generally confirmed the resulting pattern of the main analysis, although slight differences were evident. The association of having abnormal peer problems reached only marginal significance in both males and females. Moreover, in females, a significant direct association was observed between having abnormal hyperactivity/inattention with depressive symptoms (Supplementary Table S5). No clear dose–response relationship could be established due to largely overlapping confidence intervals. However, this might also be due to the reduced sample size in each group*.*

## Discussion

The present large-scale epidemiological study aimed to examine prospective associations between SDQ subscales assessed at age 10 years and the presence of depressive symptoms at age 15 years in both male and female adolescents. We found that boys and girls who exhibited emotional problems or peer problems at age 10 showed an increased risk for the presence of depressive symptoms at age 15. In addition, boys with conduct problems at age 10 were also at a greater risk of suffering from elevated depressive symptoms during adolescence.

### Prospective associations between SDQ scales and depressive symptoms

The prospective relationship between emotional problems at age 10 and depressive symptoms at age 15 is in line with our hypothesis. In this context, it needs to be highlighted that the emotional problem scale of the SDQ is closely linked to depression and includes items on depressive symptoms. In line, cross-sectional studies have shown that elevated scores on the emotional problems scale (parent-report) are associated with an increased risk of a diagnosis of a depressive disorder as assessed by standardized diagnostic instruments [10].

Moreover, parent-reported emotional problem scores on the SDQ show a low to modest correlation with depressive symptoms [59]. Thus, it is plausible that the relationship between elevated emotional problem scores at age 10 and the presence of depressive symptoms at age 15 reflects the continuity of depressive symptoms from childhood to adolescence. In this respect, our prospective findings can be brought in accordance with the results from the mental health module (BELLA study) of a German national survey (KiGGS) [10] as well as with findings from a longitudinal analysis of German health insurance data [60].

In the BELLA study, children with elevated scores on the emotional problem scale at age 9 had an increased risk of elevated scores on the same scale 6 years later [10]. Similarly, our results are consistent with prior research showing a high developmental continuity of depressive symptoms and also MD diagnoses from childhood to adolescence (e.g., [17, 61], see also [62]). Given the negative consequences of continued depressive symptoms in youth and its predictive value for a diagnosis of MD later in life [63, 64], our results emphasize the importance of early identification and prevention of depressive symptoms in youth. In this context, both indicated and universal prevention have been shown to be effective in reducing internalizing symptoms and internalizing disorders in youth (e.g. [65]), and thus represent important approaches to reduce the risk of adverse outcomes.

Our finding of the relevance of peer problems as a precursor of depressive symptoms is in accordance with prior research highlighting that problems in peer relationships play an important role in the context of depressive psychopathology. For example, longitudinal studies have demonstrated that peer victimization, which is one aspect covered by the SDQ peer problem scale, predicts depressive symptoms in adolescents (e.g., [11, 36, 37], for a meta-analysis, see [66]). It should be noted that this relationship is bidirectional, as depressive symptoms have also been shown to be an antecedent of victimization in youth [11, 36]. Given this bidirectionality, one factor which might influence the longitudinal association between peer problems and subsequent depressive symptoms is the severity of depressive symptoms during or prior to the time peer problems were evident (for conflicting results, see [37]). As the SDQ peer problem scale only includes one item that explicitly relates to peer victimization, future studies should assess this highly relevant aspect in a more comprehensive way, e.g., by applying instruments that more directly target bullying experiences in youth.

In a sensitivity analysis, we adjusted for the remaining SDQ subscales, including the emotional problem scale, which is closely related to depressive psychopathology [59]. Interestingly, the prospective association between peer problems and depressive symptoms was only slightly attenuated in boys but reached only marginal significance in girls. This sex-specific pattern can be brought in line with prior research [11] and might suggest an *independent role* of peer problems as a risk factor for depressive symptoms in boys, but not in girls. A possible explanation for the more robust prospective association between peer problems and depressive symptoms in boys compared to girls might relate to the way girls cope with peer problems. It has been found that girls talk more about peer stress and also seek more social support (e.g. from the family) in response to peer stress than boys. Such coping behaviour might buffer the effects of peer stress on internalizing psychopathology in girls (for a review, see [31]).

As expected, more boys than girls demonstrated conduct problems (e.g., [67, 68]). Moreover, we found that only in boys, conduct problems were associated with increased depressive symptoms at age 15, while this association reached only marginal significance in girls. Some previous cross-sectional studies have shown that even though girls exhibit less conduct problems than boys, those girls who show this kind of problem behaviour are at a higher risk for developing comorbid conditions, including depressive symptoms ([26, 27], but see [25]). These findings are in accordance with the so-called “gender paradox hypothesis" suggesting that the gender with the lower prevalence rate of a given psychiatric condition tends to be subjected to higher comorbidity rates [26, 69]. While at first glance, our findings seem contradictory to the gender paradox hypothesis and related findings [26, 27], it should be noted that there are no longitudinal studies supporting this hypothesis. Indeed, a large-scaled longitudinal study by Stringaris et al. [25] found an association between conduct problems at age 10 and depressive symptoms in adolescence independently of sex.

Our findings have important clinical implications. In more detail, in children and particularly in boys with conduct problems, particular attention should be given to early depressive signs to not miss early prevention and treatment opportunities [25]. Such approaches should include psychoeducation on depression for parents and children presenting with conduct problems along with practical suggestions for preventing and dealing with depressive symptoms. For example, one promising approach is the Good Behavior Game, which is a universal prevention program aiming to reduce disruptive behaviors and enhance prosocial behaviors in the classroom setting. This program has been shown to have positive long-term effects on externalizing behaviors, particularly for males exhibiting disruptive behavior early in their school career [70]. Moreover, it has also been shown to reduce suicidality in both girls and boys ([71], see [72] for a recent meta-analysis).

### Limitations

Some limitations of our study should be noted. First, while we assessed depressive symptoms based on the DesTeen at age 15, no depression-specific instrument was applied at age 10. Applying a depression screener at both time points would have allowed to also examine how cross-sectional relationships between depressive and other psychopathological symptoms change over the course of development. Second, we did not assess substance problems or substance abuse as a possible precursor to depressive symptomatology, despite research showing that substance-related psychopathology acts as a risk factor of depressive symptoms in youth [9]. However, this limitation might be of minor importance given that the present study focused on precursors evident at the age of 10, whereas considering the predictive value of substance problems or abuse seems particularly relevant during adolescence when prevalence rates of this kind of psychopathology rise [73]. Third, the parental education of most participants was high, indicating a high socioeconomic status (SES). Mental health in general [74], depression [75] and the SDQ [10, 76] are influenced by the SES in that youth with lower SES tend to have more problems, and lower SES in childhood consistently predicts worse outcomes in adolescence [9]. Thus, the strength of the associations found in this study might be underestimated due to the underrepresentation of children and adolescents with lower SES. Fourth, as in every longitudinal study, the GINIplus and LISA cohorts suffer from non-random loss to follow-up. Participants included in the analysis were more likely to have lower SDQ scores and a higher parental education level, considered as a proxy for a high socio-economic status. This might limit the generalisability of our findings. However, as higher SDQ scales are expected to be associated with lower socio-economic status [10, 76], this also might lead rather to an underestimation of the true magnitude of the effect.

### Conclusions

This study systematically examined prospective associations between different psychopathological symptom domains and depressive symptoms in adolescence, thereby also examining sex differences in these associations. Our findings highlight the relevance of identifying and targeting problems in peer relationships in late childhood as this factor was shown to predict later depressive symptoms in adolescence. Particularly in boys, it seems additionally important to consider conduct problems as a precursor of later depressive symptomatology in adolescence. Beyond these issues, our result on the association between the emotional problem scale (being closely linked to depression) and the presence of depressive symptoms corroborate earlier findings on the continuity of depressive symptomatology from childhood into adolescence across both sexes (e.g., [17]). These findings highlight the need for early detection of depressive symptoms and for the initiation of evidence-based prevention and treatment efforts in youth.

Our study offers new insight into precursors of depressive symptoms in adolescence. Given the high continuity and detrimental consequences of depressive symptoms and depressive disorders in adolescents [3, 77], identifying starting points for prevention and treatment approaches is of high clinical relevance. Our findings highlight that during late childhood, it might be of particular importance to tailor such approaches to peer and conduct problems as well as on anxious and depressive symptoms.

## Supplementary Information

Below is the link to the electronic supplementary material.

Supplementary file1 (DOCX 81 kb)

## Data Availability

Due to data protection reasons, the datasets analyzed during the current study cannot be made publicly available. The datasets are available to interested researchers from the corresponding author on reasonable request (e.g. reproducibility), provided the release is consistent with the consent given by the GINIplus and LISA study participants. Ethical approval might be obtained for the release and a data transfer agreement from the legal department of the Helmholtz Zentrum München must be accepted.
