# Supplementary material for: Psychopathological symptoms as precursors of depressive symptoms in adolescence: a prospective analysis of the GINIplus and LISA birth cohort studies
Source: Soc Psychiatry Psychiatr Epidemiol. 2022 Apr 15;57(8):1627–39. doi: 10.1007/s00127-022-02267-1 (PMC9288954; doi:10.1007/s00127-022-02267-1)
Supplement: Supplementary file 1 — Supplementary file1 (DOCX 81 kb) [file 127_2022_2267_MOESM1_ESM.docx]

**Supplementary Materials**

**Supplementary Tables**

**Supplementary Table S1: Comparison of the analysis population with the total population**

|  | **Total population (n=9085)** |  | **In analysis (n=2824)** | **p-value** |
| --- | --- | --- | --- | --- |
| **SDQ** |  |  |  |  |
| Total difficulties | 738/4860 (15.2%) |  | 379/2823 (13.4%) | <0.001 |
| Emotional problems | 871/4861 (17.9%) |  | 463/2823 (16.4%) | 0.001 |
| Conduct problems | 574/4861 (11.8%) |  | 302/2824 (10.7%) | 0.005 |
| Hyperactivity/inattention | 654/4861 (13.5%) |  | 351/2823 (12.4%) | 0.015 |
| Peer problems | 423/4860 (8.7%) |  | 218/2823 (7.7%) | 0.005 |
| Prosocial behavior | 366/4862 (7.5%) |  | 196/2823 (6.9%) | 0.069 |
| **Depressive symptoms** | 551/3989 (13.8%) |  | 389/2824 (13.8%) | 0.920 |
| **Sex [females vs. males]** | 4349/8924 (48.7%) |  | 1456/2824 (51.6%) | <0.001 |
| **Age at 15 years** | 15.2 (0.3) |  | 15.1 (0.3) | <0.001 |
| **Pubertal stage at 15 years**  **[late/post vs. early/mid pubertal]** | 2837/3626 (78.2%) |  | 2190/2824 (77.5%) | 0.059 |
| **Study center** |  |  |  | <0.001 |
| Munich | 4413/9085 (48.6%) |  | 1475/2824 (52.2%) |  |
| Leipzig | 976/9085 (10.7%) |  | 277/2824 (9.8%) |  |
| Bad Honnef | 306/9085 (3.4%) |  | 117/2824 (4.1%) |  |
| Wesel | 3390/9085 (37.3%) |  | 955/2824 (33.8%) |  |
| **Single parent** | 704/4607 (15.3%) |  | 388/2824 (13.7%) | <0.001 |
| **Parental education level** |  |  |  | <0.001 |
| low | 620/7212 (8.6%) |  | 156/2824 (5.5%) |  |
| medium | 2106/7212 (29.2%) |  | 733/2824 (26%) |  |
| high | 4486/7212 (62.2%) |  | 1935/2824 (68.5%) |  |
| **Study** |  |  |  | <0.001 |
| GINIplus observation | 3739/9085 (41.2%) |  | 1044/2824 (37%) |  |
| GINIplus intervention | 2252/9085 (24.8%) |  | 729/2824 (25.8%) |  |
| LISA | 3094/9085 (34.1%) |  | 1051/2824 (37.2%) |  |
| **Parental pathopsychology** | 522/4735 (11%) |  | 298/2796 (10.7%) | 0.346 |

*Note:* Presence of depressive symptoms at age 15 was defined as a total score ≥12 in the Depression Screener for Teenagers (DesTeen). *Abbreviation:* SDQ = Strengths and Difficulties Questionnaire.

**Supplementary Table S2: Results of logistic regression analyses additionally adjusted for parental psychopathology**

|  | **Females** | | |  | **Males** | | |
| --- | --- | --- | --- | --- | --- | --- | --- |
|  | **OR** | **95%-CI** | **p-value** |  | **OR** | **95%-CI** | **p-value** |
| Emotional problems | 1.69 | (1.20; 2.36) | 0.0025 |  | 1.86 | (1.2; 2.85) | 0.0049 |
| Conduct problems | 1.41 | (0.86; 2.24) | 0.1585 |  | 2.41 | (1.54; 3.71) | 0.0001 |
| Hyperactivity/ inattention | 1.30 | (0.78; 2.09) | 0.2976 |  | 1.29 | (0.82; 1.98) | 0.2601 |
| Peer problems | 1.75 | (1.04; 2.87) | 0.0292 |  | 2.58 | (1.56; 4.15) | 0.0001 |
| Prosocial behavior | 1.54 | (0.83; 2.73) | 0.1534 |  | 1.26 | (0.67; 2.21) | 0.4457 |

*Note*: Bonferroni-corrected P-value=0.025.

**Supplementary Table S3: Results of mutually adjusted logistic regression analysis regressing all SDQ sub-scale variables on depressive symptoms, adjusted for the confounding factors presented in Table 2**

|  | **Females** | | |  | **Males** | | |
| --- | --- | --- | --- | --- | --- | --- | --- |
|  | **OR** | **95%-CI** | **p-value** |  | **OR** | **95%-CI** | **p-value** |
| Emotional problems | 1.63 | (1.16; 2.29) | 0.0048 |  | 1.57 | (0.99; 2.45) | 0.0509 |
| Conduct problems | 1.16 | (0.69; 1.90) | 0.5565 |  | 2.13 | (1.31; 3.42) | 0.0019 |
| Hyperactivity/inattention | 1.07 | (0.62; 1.77) | 0.8049 |  | 0.89 | (0.54; 1.43) | 0.6461 |
| Peer problems | 1.58 | (0.92; 2.64) | 0.0859 |  | 2.03 | (1.18; 3.40) | 0.0081 |
| Prosocial behavior | 1.32 | (0.70; 2.35) | 0.3713 |  | 0.82 | (0.43; 1.48) | 0.5264 |

*Note*: Bonferroni-corrected P-value=0.025.

**Supplementary Table S4: Results of logistic regression analysis regressing each SDQ subscale separately on depressive symptoms (defined with cut-off ≥14), adjusted for the confounding factors presented in Table 2.**

|  | **Females** | | |  | **Males** | | |
| --- | --- | --- | --- | --- | --- | --- | --- |
|  | **OR** | **95%-CI** | **p-value** |  | **OR** | **95%-CI** | **p-value** |
| Emotional problems | 1.74 | (1.16; 2.56) | 0.0056 |  | 2.31 | (1.35; 3.83) | 0.0016 |
| Conduct problems | 1.41 | (0.78; 2.39) | 0.2279 |  | 1.99 | (1.12; 3.42) | 0.0150 |
| Hyperactivity/inattention | 1.14 | (0.60; 2.01) | 0.6686 |  | 1.94 | (1.14; 3.21) | 0.0121 |
| Peer problems | 2.16 | (1.21; 3.68) | 0.0065 |  | 2.45 | (1.31; 4.35) | 0.0032 |
| Prosocial behavior | 1.51 | (0.72; 2.89) | 0.2387 |  | 1.14 | (0.51; 2.28) | 0.7222 |

*Note*: Bonferroni-corrected P-value=0.025.

**Supplementary Table S5. Results of logistic regression analyses using a three-level categorization of SDQ scales**

|  |  | Females | | |  | Males | | |
| --- | --- | --- | --- | --- | --- | --- | --- | --- |
|  |  | OR | 95%-CI | p-value |  | OR | 95%-CI | p-value |
| Emotional problems | Borderline vs. Normal | 1.90 | (1.20; 2.95) | 0.005 |  | 2.13 | (1.12; 3.81) | 0.015 |
|  | Abnormal vs. Normal | 1.65 | (1.05; 2.53) | 0.024 |  | 1.90 | (1.10; 3.16) | 0.017 |
| Conduct problems | Borderline vs. Normal | 1.64 | (0.92; 2.83) | 0.082 |  | 2.52 | (1.46; 4.21) | 0.001 |
|  | Abnormal vs. Normal | 1.35 | (0.62; 2.69) | 0.411 |  | 2.46 | (1.25; 4.54) | 0.006 |
| Hyperactivity/  inattention | Borderline vs. Normal | 0.93 | (0.40; 1.92) | 0.859 |  | 1.29 | (0.62; 2.44) | 0.464 |
|  | Abnormal vs. Normal | 1.87 | (1.01; 3.31) | 0.037 |  | 1.43 | (0.83; 2.35) | 0.181 |
| Peer problems | Borderline vs. Normal | 2.01 | (1.00; 3.82) | 0.040 |  | 3.56 | (1.87; 6.49) | <0.001 |
|  | Abnormal vs. Normal | 1.81 | (0.88; 3.50) | 0.091 |  | 1.88 | (0.90; 3.64) | 0.074 |
| Prosocial behavior | Borderline vs. Normal | 1.90 | (0.91; 3.73) | 0.074 |  | 0.89 | (0.37; 1.88) | 0.786 |
|  | Abnormal vs. Normal | 0.86 | (0.25; 2.29) | 0.784 |  | 1.81 | (0.75; 3.89) | 0.154 |

**Supplementary Figure**

**Supplementary Figure S6: Flow chart illustrating sample selection for the analysis presented in the manuscript text.**

*Abbrevations:* SDQ = Strengths and Difficulties Questionnaire; DesTeen = Depression Screener for Teenagers.
